# Prognostic Importance of the Preoperative Naples Prognostic Score for Patients With Adenocarcinoma of the Esophagogastric Junction

**DOI:** 10.3389/fonc.2020.595793

**Published:** 2020-12-16

**Authors:** Jianping Xiong, Yaqin Wang, Wenzhe Kang, Fuhai Ma, Hao Liu, Shuai Ma, Yang Li, Peng Jin, Haitao Hu, Yantao Tian

**Affiliations:** ^1^ Department of Pancreatic and Gastric Surgery, National Cancer Center/National Clinical Research Center for Cancer/Cancer Hospital, Chinese Academy of Medical Sciences and Peking Union Medical College, Beijing, China; ^2^ Department of Interventional Radiology, The First Affiliated Hospital of China Medical University, Shenyang, China

**Keywords:** adenocarcinoma of esophagogastric junction, naples prognostic score, time-dependent receiver operating characteristic, prognosis, prognostic factors

## Abstract

**Background:**

The naples prognostic score (NPS) is established according to nutritional or inflammatory state, and it is identified as the new prognostic score for a variety of malignant tumors. However, its significance in cases suffering from adenocarcinoma of esophagogastric junction (AEJ) who receive surgery remains unclear so far.

**Methods:**

In this study, patients receiving surgery without preoperative therapy were examined between June 2007 and August 2017 in a retrospective way. Typically, the serum albumin level, total cholesterol level, neutrophil-to-lymphocyte ratio, together with the lymphocyte-to-monocyte ratio, was determined to calculate the NPS. The prognostic impact of NPS was evaluated using survival analyses. Time-dependent receiver operating characteristic curve (t-ROC) analysis was also carried out for comparing prognostic impacts of those scoring systems.

**Results:**

Altogether 231 cases were enrolled in this study. A higher NPS showed positive correlation with perineural invasion. Upon multivariate analysis, NPS was identified to be the independent prognostic factor to predict overall survival (OS) along with relapse-free survival (RFS) (both P< 0.05), and an especially strong correlation was observed at advanced pTNM stages based on NPS system. As for subgroup analyses on adjuvant chemotherapy or surgery only, NPS still independently predicted the OS as well as RFS (both P< 0.05) in both groups. Furthermore, t-ROC analysis showed that NPS was more accurate than the systemic inflammation score in predicting OS and RFS.

**Conclusions:**

The NPS represents the simple and useful rating system, which can independently predict the survival for AEJ cases undergoing surgery.

## Introduction

Adenocarcinoma of esophagogastric junction (AEJ) represents adenocarcinoma that has the epicenter of less than 5 cm away from esophagogastric junction by the World Health Organization (WHO) (1–3). AEJs were classified into three subtypes (type I, type II, and type III) according to Siewert’s classification (1). Type I denotes adenocarcinoma of the distal esophagus with an epicenter located between 1 and 5 cm above the esophagogastric junction (EGJ). Type II denotes true carcinoma of the cardia with a tumor epicenter between 1cm above and 2cm below the EGJ. Type III denotes subcardial carcinoma with a tumor epicenter between 2 and 5 cm below the EGJ. The incidence of AEJ has increased rapidly in most countries over the last few decades (4). For instance, Devesa et al. reported that the AEJ incidence rate increased by 20%, and that elevated by 3 to 4 times in patients aged over 65 years in 1998 (5). AEJ is an aggressive malignancy, with the 5-year survival after diagnosis being less than 20% (6). In addition, an increasing number of studies have shown that AEJ should be considered separately from esophageal cancer (EC) or gastric cancer (GC) because of its unique clinicopathological characteristics and survival outcome. Systemic inflammation exerts a vital part during cancer genesis and development; besides, the systemic inflammation markers are related to the prognosis for tumor patients (7, 8). The inflammatory biomarker concentrations in serum before treatment, including lymphocyte-to-monocyte ratio (LMR), platelet-to-lymphocyte ratio (PLR), or neutrophil-to-lymphocyte ratio (NLR), have been found to be related to the progression and prognostic outcomes of many cancer types, including GC, hepatocellular carcinoma (HCC), and EC (9, 10). Moreover, preoperative serum albumin (Alb) concentration has also been used to predict the survival outcomes for lung cancer (LC), GC and EC patients (11, 12). Recently, Galizia et al. first reported that the Naples prognostic score (NPS) was the novel rating system formulated according to serum Alb level, total cholesterol (TC) level, NLR along with LMR, which reflected the nutritional and systemic inflammatory statuses in cancer patients (13). Typically, the NPS is strongly associated with the prognostic outcomes for colorectal cancer (CRC), pancreatic cancer, lung cancer, gastric cancer, and osteosarcoma (13–17). Additionally, studies have shown that the NPS is more accurate than other prognostic factors in predicting survival (13, 14, 18). So far, few researches has been carried out to examine the role of NPS in predicting the prognosis for AEJ cases receiving surgical resection. Only one retrospective study from Italian detected a significant association between NPS and outcome in patients undergoing proximal gastric cancers surgery (17).

Therefore, the present retrospective cohort study was conducted aiming to determine the effect of NPS on predicting the prognosis for AEJ patients, and to investigate the relationships between NPS and other clinicopathological features.

## Patients and Methods

### Study Population

This study retrospectively assessed each case undergoing radical surgery for AEJ from June 2007 to August 2017 at the Department of Pancreatic and Gastric Surgery of the National Cancer Center/Cancer Hospital, Chinese Academy of Medical Sciences and Peking Union Medical College. The following exclusion criteria were applied: (1) palliative surgery, (2) no routine blood examination before surgery, (3) distant metastasis at the time of surgery, (4) neoadjuvant chemotherapy, (5) malignant disease in other organs, (6) emergency operation, (7) other concurrent malignancies, (8) R1/R2 resection, (9) incomplete/inaccurate medical records, (10) chronic liver and/or kidney diseases, (11) missing laboratory data, (12) missing follow-up data, and (13) < 3 months of follow-up period. Finally, 231 cases were included into the present work. The demographic, histopathological, along with laboratory variables for all patients were retrospectively analyzed, and relevant data were extracted from our hospital database and patient records. Clinical tumor stage was determined in line with the Pathological Tumor Lymph Node Metastasis (pTNM) Classification released by the International Union for Cancer Control (UICC) (7th edition). Meanwhile, the appropriate surgical procedure was selected according to the location of AEJ (namely, abdominothoracic enbloc esophagectomy or transhiatal extended gastrectomy). The treatment for each patient was discussed by a multidisciplinary team of oncology before surgery. Oxaliplatin/capecitabine or cisplatin/5-fluorouracil was recommended as the adjuvant chemotherapy regimen for advanced AEJ patients.

### Definition of Inflammation-Based Indicators

Routine blood test was performed at a week before surgery. All the blood test results conducted at a week before surgery were acquired from the Laboratory Database of National Cancer Center (Beijing, China). No patient developed sign of pyrexia (axillary temperature ≥ 37.2°C/99.0°F) or chronic inflammation or active infection. For all patients, preoperative data were collected, including body mass index (BMI), tumor size, ASA score, serum Alb concentration, TC level, absolute neutrophil count, absolute monocyte count, along with absolute lymphocyte count. In addition, the systemic inflammation score (SIS) was defined as follows: the scores were 2 for cases having serum Alb concentration < 40 g/L and LMR <4.44; the scores were 1 for cases having serum Alb concentration ≥ 40 g/l or LMR ≥ 4.44; and the scores were 0 for cases having both serum Alb concentration ≥ 40 g/l and LMR ≥ 4.44. According to Galizia et al’s method, the serum Alb concentration, TC level, NLR together with LMR were determined to calculate NPS (13) (**Supplemental Figure 1**).

### Follow-Up Analysis

The follows-up visits after surgery were evaluated at an interval of 3 months within 2 years after surgery, and 6 months thereafter. The last follow-up was assessed in October 2019. Follow-up examinations included tumor markers (CEA, CA19-9 and CA72-4), chest X-ray, abdominopelvic computed tomography (CT), and annual endoscopy. In this study, overall survival (OS), which indicated the duration between surgical resection and all-cause death or the final follow-up, was adopted as the primary endpoint. In addition, relapse-free survival (RFS), which represents the time between surgery and relapse or death, was used as the secondary endpoint. Death from any cause was considered as an event.

### Statistical Methods

Categorical variables were analyzed by chi-square test, while continuous variables were analyzed by the *t-*tests. The Kaplan-Meier (KM) method was applied in plotting survival curve, whereas differences between the curves were analyzed by log-rank test. The significant variables selected from univariate analysis were incorporated into multivariate analysis according to the Cox regression analysis. In addition, the time-dependent receiver operating characteristic (t-ROC) curves, together with predicted values of area under the curve (AUC), were adopted for comparing the significance of SIS and NPS in prognosis prediction (19). Each test was bilateral, and a difference of P<0.05 indicated statistical significance. The SPSS 18.0 (SPSS Inc., Chicago, IL, USA) and R ver. 4.0.2 (R Foundation for Statistical Computing, Vienna, Austria) were employed for statistical analysis. Additionally, R package “rms” was used for calculating the C index, and t-ROC analysis was performed using the R package “timeROC”.

## Results

### Patient Characteristics

Totally, 231 AEJ cases were enrolled into the present work (**Supplemental Figure 2**), including 200 (86.6%) male and 31 (13.4%) female patients. The average age at surgery of these patients was 57.8 (range, 43.4–77.9) years. According to the pTNM staging system, 48 (20.8%) patients were at stage I, 71 (30.7%) at stage II, and 112 (48.5%) at stage III, respectively. 11 had type I (4.9%) AEJs, 135 had type II (58.6%), and 85 had type III (36.5%). Of those 231 cases, 129 received adjuvant chemotherapy. According to NPS system, 44 patients (19.1%) were assigned into group 0 (NPS 0), 150 (64.9%) in group 1 (NPS 1 or 2), and 37 (16.0%) in group 2 (NPS 3 or 4).

### Relationships of Preoperative NPS System With Clinicopathological Features

**Table 1** summarizes relationships of NPS with clinicopathological features. As observed from the table, NPS values remarkably increased among patients suffering from perineural invasion (P = 0.003). However, no significant difference was observed in age, BMI, ASA score, tumor size, tumor differentiation, vascular involvement, pTNM stage, surgical approach, or adjuvant chemotherapy among these three NPS groups. In addition, the NPS remarkably increased among cases having the serum Alb (mg/dl) < 40 (P< 0.001), TC (mg/dl) ≤ 180 (P< 0.001), LMR ≤ 4.44 (P< 0.001), while NLR > 2.96 (P< 0.001).

**Table 1:** Association of NPS and clinicopathological characteristics in patients with AEJ.

Clinicopathological features	All cases	Group 0 (n = 44)	Group 1 (n = 150)	Group 2 (n = 37)	P value
Age (median)	62.2	61.0	61.8	65.4	0.701
Gender Male Female	200 (86.6) 31 (13.4)	35 (79.5) 9 (20.5)	132 (88.0) 18 (12.0)	33 (89.2) 4 (10.8)	0.439
BMI (median)	24.3	23.7	24.7	23.5	0.883
ASA score 1 2 3	14 (6.1) 183 (79.2) 34 (14.7)	2 (4.5) 38 (86.4) 4 (9.1)	10 (6.7) 116 (77.3) 24 (16.0)	2 (5.4) 29 (78.4) 6 (16.2)	0.332
Tumor size (cm, median)	4.4	4.2	4.6	5.7	0.206
Siewert classification Type I Type II Type III	11 (4.9) 135 (58.6) 85 (36.5)	2 (4.0) 25 (57.7) 17 (38.3)	8 (5.2) 86 (57.3) 56 (37.5)	1 (2.7) 24 (64.8) 12 (32.5)	0.241
cTNM stage I II III	42 (18.1) 68 (29.4) 121 (52.5)	10 (22.7) 12 (27.3) 22 (50.0)	27 (18.0) 47 (31.3) 76 (50.7)	5 (13.5) 9 (24.3) 23 (62.2)	0.173
Tumor differentiation G1 G2 G3	19 (8.2) 135 (58.4) 77 (33.4)	6 (13.6) 26 (59.1) 12 (27.3)	10 (6.7) 91 (60.7) 49 (32.6)	3 (8.1) 18 (48.6) 16 (43.3)	0.103
Vascular invasion Negative Positive	149 (64.5) 82 (35.5)	22 (50.0) 22 (50.0)	95 (63.3) 55 (36.7)	23 (62.2) 14 (37.8)	0.273
Perineural invasion Negative Positive	101 (43.7) 130 (56.3)	32 (72.7) 12 (27.3)	64 (42.7) 86 (57.3)	15 (40.5) 22 (59.5)	0.003
Surgical approach Abdominal Thoracoabdominal	48 (20.8) 183 (79.2)	13 (29.5) 31 (70.5)	30 (20.0) 120 (80.0)	5 (13.5) 32 (86.5)	0.084
pTNM stage I II III	48 (20.8) 71 (30.7) 112 (48.5)	12 (27.3) 13 (29.5) 19 (43.2)	30 (20.0) 47 (31.3) 73 (48.7)	6 (16.2) 11 (29.7) 20 (54.1)	0.329
Adjuvant chemotherapy No Yes	102 (44.2) 129 (55.8)	21 (47.7) 23 (52.3)	66 (44.0) 84 (56.0)	15 (40.5) 22 (59.5)	0.517
Serum albumin (mg/dl) ≥ 40 < 40	193 (83.5) 38 (16.5)	44 (100.0) 0 (0)	135 (90.0) 15 (10.0)	14 (37.8) 23 (62.2)	< 0.001
Total cholesterol (mg/dl) >180 ≤180	98 (42.4) 133 (57.6)	42 (55.5) 2 (45.5)	50 (33.3) 100 (66.7)	6 (16.4) 31 (83.8)	< 0.001
Neutrophil: lymphocyte ratio ≤2.96 >2.96	189 (81.8) 42 (18.2)	44 (100) 0 (0)	134 (89.3) 16 (10.7)	11 (29.7) 26 (70.3)	< 0.001
Lymphocyte: monocyte ratio >4.44 ≤4.44	111 (48.1) 120 (51.9)	43 (97.7) 1 (2.3)	67 (44.7) 83 (55.3)	1 (7.7) 36 (92.3)	< 0.001

AEG, adenocarcinoma of the gastroesophageal junction; NPS, naples prognostic score; BMI, body mass index.

### OS and RFS Examined on the Basis of NPS

**Figure 1:**
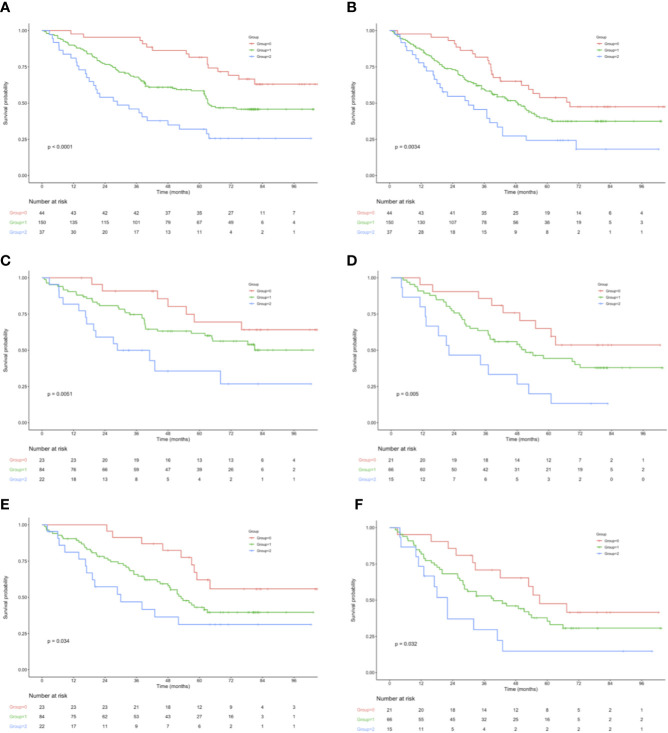
**(A)** OS in 44 patients (group 0), 150 patients (group 1), and 37 patients (group 2), who underwent surgery for AEJ. **(B)** RFS in 44 patients (group 0), 150 patients (group 1), and 37 patients (group 2), who underwent surgery for AEJ. **(C)** Association of the NPS with the OS in the adjuvant chemotherapy group. **(D)** Association of the NPS with the OS in the surgery alone group. **(E)** Association of the NPS with the RFS in the adjuvant chemotherapy group. **(F)** Association of the NPS with the RFS in the surgery alone group. OS, overall survival. RFS, relapse-free survival. AEG, adenocarcinoma of the gastroesophageal junction. NPS, naples prognostic score. SIS, systemic inflammation score.

**Figure 2:**
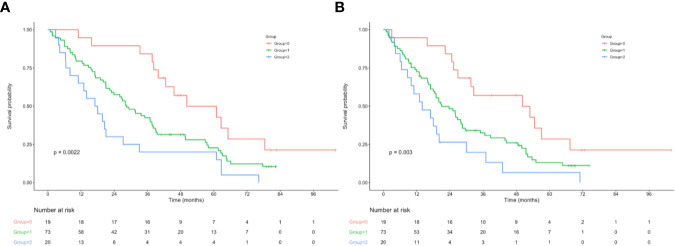
**(A)** Relationship between NPS and the OS of patients with stage III AEJ. **(B)** Relationship between NPS and the RFS of patients with stage III AEJ. OS, overall survival; RFS, relapse-free survival; AEG, adenocarcinoma of the gastroesophageal junction; NPS, naples prognostic score.

OS and RFS curves were statistically analyzed, as shown in **Figure 1**. The OS rates at 1, 3, and 5 years were 90.0%, 68.8%, and 43.3%, respectively, for those included cases, with the median OS and RFS of 49.5 and 43.6 months, respectively, for those included cases. With regard to OS, the median OS was 60.0, 49.2, and 43.6 months for groups 0, 1, and 2, respectively; whereas the median RFS was 56.4, 43.6, and 32.4 months for the above three groups, respectively. The KM survival analysis indicated that, a high NPS was related to poor OS and RFS for all included patients (**Figures 1A, B** **)**. Furthermore, significant difference was observed in OS based on the NPS in surgery alone group and postoperative adjuvant chemotherapy group (**Figures 1C–F**). However, when stratified by pTNM stage, the most significant differences in OS and RFS were observed in the stage III subgroup based on NPS system (**Figure 2**, **Supplemental Figure 3**).

### Univariate Together With Multivariate Analyses on the Prognostic Predictors Among AEJ Cases

On the one hand, univariate analysis and multivariate analyses both revealed that pTNM stage (II: HR = 1.66, P< 0.001; III: HR = 4.29, P< 0.001), tumor size (HR = 1.62, P< 0.001), tumor differentiation (G2: HR = 1.42, P = 0.005; G3: HR = 2.02, P< 0.001), adjuvant chemotherapy (HR = 0.60, P< 0.001), vascular invasion (HR = 1.86, P< 0.001), and perineural invasion (HR = 1.91, P= 0.001) were associated with OS (**Table 2**). In addition, OS was markedly impaired among cases having serum Alb level (mg/dl) < 40 (HR = 0.68, P= 0.002), LMR ≤ 4.44 (HR = 1.77, P< 0.001), together with NLR> 2.96 (HR = 1.58, P = 0.022) (**Table 2**, **Supplemental Figure 4**). Upon multivariate analyses, SIS was also a significant prognostic factor for OS (SIS= 1: HR = 1.53, P = 0.003; SIS= 2: HR = 2.12, P = 0.016) (**Table 2**, **Supplemental Figure 5**). On the other hand, univariate analysis determined that the following factors were the important prognostic factors for RFS, including pTNM stage (II: HR = 2.19, P = 0.002; III: HR = 4.16, P = 0.008), tumor size (HR = 1.71, P= 0.005), tumor differentiation (G2: HR = 1.49, P = 0.020; G3: HR = 2.43, P = 0.003), perineural invasion (HR = 2.48, P = 0.002), vascular invasion (HR = 2.13, P = 0.001), adjuvant chemotherapy (HR = 0.79, P< 0.001), serum Alb (HR = 0.69, P< 0.001), NLR (HR = 2.33, P = 0.007), as well as LMR (HR = 1.45, 95% CI = 1.16–2.93, P = 0.003) (**Table 3**). As suggested by multivariate analysis, pTNM stage (II: HR = 1.70, III: HR = 3.53, P< 0.001), tumor size (HR = 1.35, P< 0.001), tumor differentiation (G2: HR = 1.36, P = 0.009; G3: HR = 2.27, P< 0.001), vascular invasion (HR = 1.74, P< 0.001), perineural invasion (HR = 1.80, P< 0.001), adjuvant chemotherapy (HR = 0.66, P< 0.001), NLR (HR = 1.82, P = 0.015), along with LMR (HR = 1.28, P = 0.007) were identified as the predictors to independently predict RFS (**Table 3**, **Supplemental Figure 6**). Although the SIS was identified as the independent factor to predict RFS, the HR of SIS= 1 and SIS= 2 were similar, revealing no dose- responsibility (SIS= 1: HR = 1.98, P = 0.031; SIS= 2: HR = 2.01, P = 0.009) (**Table 3**, **Supplemental Figure 5**).

**Table 2:** Univariate and multivariate analysis of clinicopathologic variables in relation to OS in patients with AEJ.

Clinicopathological features	Univariate analysis	P value	Multivariate analysis	P value
Age	1.33 (0.85, 1.74)	0.209		
Gender Male Female	Reference 0.82 (0.56–1.16)	0.462		
BMI	0.73 (0.49, 1.45)	0.197		
ASA score 1 2 3	Reference 1.33 (0.76, 1.92) 1.07 (0.54, 1.68)	0.265 0.420		
Tumor size (cm)	2.09 (1.41, 2.83)	< 0.001	1.62 (1.25, 2.19)	< 0.001
Siewert classification Type I Type II Type III	Reference 0.75 (0.43, 1.37) 0.81 (0.54, 1.64)	0.249 0.810		
cTNM stage I II III	Reference 1.72 (1.22–2.34) 4.89 (2.61, 7.71)	0.002 0.001	Reference 1.61 (1.18–2.02) 4.10 (2.11, 5.89)	< 0.001 < 0.001
Tumor differentiation G1 G2 G3	Reference 1.65 (1.21, 2.17) 2.13 (1.40, 3.25)	0.012 0.001	Reference 1.42 (1.15, 1.88) 2.02 (1.26, 3.01)	0.005 < 0.001
Vascular invasion Negative Positive	Reference 1.67 (1.31–1.85)	< 0.001	Reference 1.86 (1.37–2.13)	< 0.001
Perineural invasion Negative Positive	Reference 1.83 (1.26–2.10)	< 0.001	Reference 1.91 (1.35–2.47)	0.001
Surgical approach Abdominal Thoracoabdominal	1.21 (0.83, 1.52)	0.117		
pTNM stage I II III	Reference 1.87 (1.26–2.62) 5.01 (2.93, 8.62)	0.005 0.003	Reference 1.66 (1.21–2.33) 4.29 (2.18, 6.55)	< 0.001 < 0.001
Adjuvant chemotherapy No Yes	Reference 0.63 (0.42, 0.78)	< 0.001	Reference 0.60 (0.31, 0.74)	< 0.001
Serum albumin (mg/dl) <40 ≥40	Reference 0.74 (0.53, 0.98)	< 0.001	Reference 0.68 (0.45, 0.91)	0.002
Total cholesterol (mg/dl) >180 ≤180	Reference 1.81 (0.45, 2.96)	0.074		
Neutrophil: lymphocyte ratio ≤2.96 >2.96	Reference 1.64 (1.10, 2.35)	0.003	Reference 1.58 (1.12, 2.47)	0.022
Lymphocyte: monocyte ratio >4.44 ≤4.44	Reference 1.82 (1.23, 2.19)	< 0.001	Reference 1.77 (1.20, 2.03)	0.001
SIS 0 1 2	Reference 1.42 (1.15, 1.86) 1.78 (1.31, 2.82)	0.009 < 0.001	Reference 1.53 (1.13, 3.16) 2.12 (1.24, 4.84)	0.003 0.016
NPS 0 1 2	Reference 2.12 (1.41, 2.97) 3.45 (2.31, 3.38)	< 0.001 < 0.001	Reference 1.85 (1.22, 2.43) 3.29 (2.16, 3.17)	< 0.001 < 0.001

OS, overall survival. AEG, adenocarcinoma of the gastroesophageal junction. BMI, body mass index. NPS, naples prognostic score. SIS, systemic inflammation score.

**Table 3:** Univariate and multivariate analysis of clinicopathologic variables in relation to RFS in patients with AEJ.

Clinicopathological features	Univariate analysis	P value	Multivariate analysis	P value
Age	1.12 (0.83, 1.36)	0.335		
Gender Male Female	Reference 0.72 (0.41–1.35)	0.733		
BMI	0.89 (0.56, 1.28)	0.185		
ASA score 1 2 3	Reference 1.44 (0.53, 2.37) 1.38 (0.75, 2.02)	0.303 0.220		
Tumor size (cm)	1.71 (1.20, 3.34)	0.005	1.35 (1.12, 1.94)	< 0.001
Siewert classification Type I Type II Type III	Reference 0.68 (0.41, 1.35) 0.84 (0.52, 1.73)	0.201 0.729		
cTNM stage I II III	Reference 1.56 (1.20–2.62) 4.61 (2.38, 6.90)	0.008 0.003	Reference 1.49 (1.17–2.18) 4.02 (2.09, 5.34)	< 0.001 < 0.001
Tumor differentiation G1 G2 G3	Reference 1.49 (1.18, 2.35) 2.43 (1.62, 3.44)	0.020 0.003	Reference 1.36 (1.12, 1.90) 2.27 (1.35, 3.18)	0.009 < 0.001
Vascular invasion Negative Positive	Reference 2.13 (1.43–3.14)	0.001	Reference 1.74 (1.21–2.05)	< 0.001
Perineural invasion Negative Positive	Reference 2.48 (1.31–4.68)	0.002	Reference 1.80 (1.15–2.11)	< 0.001
Surgical approach Abdominal Thoracoabdominal	1.62 (0.77, 2.38)	0.168		
pTNM stage I II III	Reference 2.19 (1.35–2.43) 4.16 (2.02, 5.36)	0.002 0.008	Reference 1.70 (1.26–2.62) 3.53 (1.66, 5.27)	< 0.001 < 0.001
Adjuvant chemotherapy No Yes	Reference 0.79 (0.46, 0.83)	< 0.001	Reference 0.66 (0.35, 0.80)	< 0.001
Serum albumin (mg/dl) < 40 ≥ 40	Reference 0.69 (0.33, 0.82)	< 0.001	Reference 0.71 (0.40, 1.85)	0.077
Total cholesterol (mg/dl) < 180 ≥ 180	Reference 1.96 (0.27, 3.73)	0.250		
Neutrophil: lymphocyte ratio < 2.96 ≥ 2.96	Reference 2.33 (1.48, 4.78)	0.007	Reference 1.82 (1.36, 3.64)	0.015
Lymphocyte: monocyte ratio < 4.44 ≥ 4.44	Reference 1.45 (1.16, 2.93)	0.003	Reference 1.28 (1.09, 1.87)	0.007
SIS 0 1 2	Reference 2.65 (1.17, 4.80) 3.02 (1.64, 6.91)	0.010 0.008	Reference 1.98 (1.12, 3.19) 2.01 (1.34, 7.18)	0.031 0.009
NPS 0 1 2	Reference 2.85 (1.34, 4.13) 4.07 (1.79, 6.52)	0.001 0.015	Reference 2.28 (1.46, 3.11) 3.40 (2.04, 4.36)	< 0.001 < 0.001

RFS, relapse-free survival; AEG, adenocarcinoma of the gastroesophageal junction; BMI, body mass index; NPS, naples prognostic score; SIS, systemic inflammation score.

### Prognostic Value of NPS

**Figure 3:**
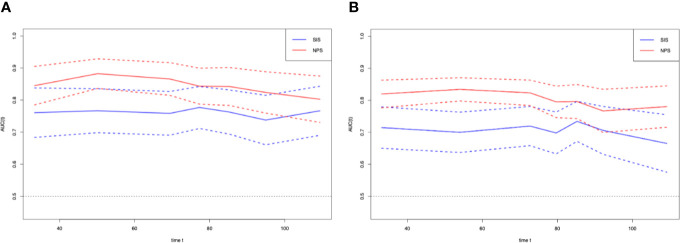
Time-dependent ROC curves for the NPS and SIS. The horizontal axis represents year after surgery, and the vertical axis represents the estimated AUC for survival at the time of interest. Red and blue solid lines represent the estimated AUCs for the NPS and SIS, respectively, and broken lines represent the 95% confidence intervals for each AUC. **(A)** overall survival. **(B)** relapse-free survival. NPS, naples prognostic score. SIS, systemic inflammation score; AUC, area under the curve; ROC, receiver operating characteristic.

T-ROC curves were established for comparing prognostic values between SIS and NPS (**Figure 3**). During the entire observation period, the t-ROC curve of NPS continued to outperform that of SIS. Furthermore, when evaluating the prediction performances of NPS and SIS in predicting OS, the AUC values of SIS were significantly lower than those of NPS at 1, 3, and 5 years after surgery (1 year: 0.775 vs. 0.863, P = 0.013; 3 years: 0.7760.871 vs. 0.871, P = 0.009; 5 years: 0.679 vs. 0.869, P = 0.002). Also, NPS displayed apparently great accuracy compared with SIS in predicting RFS.

## Discussion

The present work examined the values of NPS in predicting the prognosis for AEJ patients who underwent radical resection. Our study indicated that the NPS was strongly associated with both OS and RFS, and patients with higher NPS had decreased OS and RFS.

Since the first systemic report of the relationship inflammation with cancer by Virchow in the 19th century, an increasing number of studies have shown that systemic inflammation is an important part of TME (7, 20). Growing evidence indicates that the inflammatory reaction in microenvironment contributes to tumor progression, including the induction of angiogenesis, tumor cell proliferation and metastasis (21). As suggested in plenty of studies, the inflammation-related prognostic scores, including LMR, NLR, and PLR, are associated with the prognosis for various malignant tumors, including GC, HCC, and esophageal squamous cell carcinoma (ESCC) (22, 23). However, the host’s situation can affect the prognostic abilities of a single inflammation-related marker, and a single marker is potentially misguiding in the presence of randomly determined threshold. Recently, an increasing number of studies report that NPS, which is established on the basis of preoperative serum Alb concentration, TC level, LMR together with NLR, is a novel inflammation-based prognostic score. Researchers have identified that NPS has prognostic value for pancreatic cancer, CRC and osteosarcoma (13, 14, 16). It takes into account the effects of both nutritional status and systemic inflammation on tumor prognosis. Therefore, NPS is superior to other single inflammatory or nutritional related markers.

More and more studies have determined the relationships of systemic inflammation and malnutrition with tumorigenesis, tumor growth, tumor metastasis and tumor progression, and they are confirmed within various cancer types, such as GC and EC. As a result, more efforts are made to identify the inflammation- and nutrition-related markers and to develop a novel prognostic scoring system. In particular, the reduction in serum Alb concentration is not only a sign of malnutrition, but also a sign of systemic inflammation, since serum Alb content is reduced *via* certain proinflammatory factors like cytokines (24). Alb is a serum protein with greatest abundance, which has the ability to stabilize cell growth and DNA replication, maintain diverse biochemical variations, and play an important role of antioxidant in carcinogens (25). Hence, the reduction in serum Alb concentration not only reflects hepatic insufficiency, but also indicates the lack of human defense capabilities, like cellular immunity and humoral immunity, which may thus increase the possibility of human infection and lead to poor response to anti-cancer treatment (26). Serum Alb concentration is currently included in most scoring systems. Hyperproteinemia is associated with better survival in AEJ, as observed in the present study. Low cholesterol level has been reported to link with poor prognosis for many human tumors, such as GC, EC and renal cell carcinoma (RCC) (27, 28). Hypocholesterolemia affects the liquidity of cellular membrane, which thus decreases cell surface receptor mobility, together with the capacity of transmembrane signal transmission (29). Consequently, immunocompetent cells can not destroy cancer cells due to the changes in their membranes (30). Our study found that, the low cholesterol levels were associated with poor survival, but there was no significant difference. LMR consists of lymphocytes and monocytes, whereas NLR is constituted by neutrophils and lymphocytes. Lymphocytes represent the fundamental part in the intrinsic and adaptive immune systems, as well as the cell foundation for immune editing and surveillance (31). Lymphocytes can enhance the immune surveillance ability of cancer, thereby inhibiting the proliferation, invasion, and metastasis of tumor cells (31). As reported, tumor-infiltrating lymphocytes are associated with improved prognosis for various cancers, which may be attributed to the anti-tumor activity and angiogenesis inhibition induced by tumor-infiltrating lymphocytes (32). Therefore, lymphopenia is associated with poor prognostic outcomes for cancer cases. As reported in previous works, the circulatory monocytes facilitate cancer growth and decrease the immune monitoring (33). In addition, studies have shown that monocytes may promote the metastasis of tumor cells through the tumor-monocyte-endothelial interaction (34). The anti-tumor activity of cytotoxic CD8 T cells may be inhibited by the increasing peri-tumorial neutrophil count, resulting in tumor growth and metastasis (35). In addition, in patients with higher NLR, the cytokines released by neutrophils, including interleukin-18 (IL-18), vascular endothelial growth factor (VEGF), together with matrix metalloproteinases (MMP), may contribute to tumor growth (36). Similar to prior works, the present work showed that both LMR and NLR greatly affected AEJ prognosis. The multivariate analysis indicated that both LMR and NLR was the factor to independently predict OS and RFS.

NPS indicates both inflammatory and nutritional states, along with SIS. Typically, SIS, which is based on the preoperative serum Alb concentration and LMR, is considered as a novel prognostic indicator for GC and ESCC (37, 38). The results of our study suggested that SIS was related to the prognosis for AEJ patients; But when we use the SIS to predict RFS, the HR of SIS=1 is similar to the HR of SIS=2, and there is no dose responsibility. Besides, this study also compared the prognostic values of the NPS with SIS. The t-ROC curve analysis using NPS and SIS for predicting OS and RFS indicated a trend that, the AUC for NPS was higher than that for SIS. The NPS exhibited significantly superior accuracy SIS in predicting OS and RFS.

Compared with the existing tools to target immunonutritional interventions, our system has the major strength that, by combining the oncological, nutritional, and immunological parameters, it outperforms the existing nutritional indices in predicting the postoperative adverse events; besides, it targets the immunonutritional intervention to patients who may benefit the most. The results of our study indicated that early inflammation control and nutritional support might improve the prognosis for cancer patients. In addition, preoperative identification of patient status could have several uses in clinical practice, including prognostic stratification and treatment. Early detection and improvement of malnutrition and inflammation may result in better patient outcomes (24).

Certain limitations should be noted in this study. Firstly, selection bias was inevitable due to the retrospective nature, even though samples were selected in strict accordance with inclusion and exclusion standards. Secondly, patients who received NACT were eliminated from this study, yet it was difficult to guarantee the identical patient status prior to blood sample collection, and our findings in the present work did not apply to AEJ cases after NACT. Thirdly, according to the 7th UICC system, a tumor whose epicenter is within the proximal 5 cm of the stomach and that extends into the EGJ is now regarded and staged as esophageal cancer (39); however, due to the different staging of esophageal cancer and gastric cancer, some cases have caused tumor staging drift, which affects the formulation of postoperative treatment plans. This study decided to compare the NPS with the 7th TNM instead of the last available TNM and therefore these data could be less interesting for the scientifical community.

## Conclusions

To sum up, this work suggests that preoperative NPS can serve as a simple and useful predictor to predict AEJ prognosis. Besides, NPS is also utilized as a part of the preoperative prognostic stratification and the post-operative follow-up, so as to develop the individualized treatment for AEJ patients.

## Data Availability Statement

The original contributions presented in the study are included in the article/**Supplementary Material**. Further inquiries can be directed to the corresponding author.

## Ethics Statement

The studies involving human participants were reviewed and approved by Institutional Review Board of Cancer Hospital, Chinese Academy of Medical Sciences and Peking Union Medical College. The patients/participants provided their written informed consent to participate in this study.

## Author Contributions

JX conceived the study and wrote the manuscript. WK, HH, and FM searched the database, reviewed the studies, and collected the data. YW, HL, and SM performed the statistical analyses. YL and PJ performed revision of the manuscript. YT arranged for and provided the funding for this work. All authors reviewed the manuscript and participated in its revision. YT is the guarantor for this study. JX, YW, and WK contributed equally to this work. All authors contributed to the article and approved the submitted version.

## Funding

This work was supported by grants from the National Natural Science Foundation of China (81772642), the Beijing Municipal Science & Technology Commission (Z161100000116045), and Capital’s Funds for Health Improvement and Research (CFH 2018-2-4022). The funders had no role in study design, data collection and analysis, decision to publish, or preparation of the manuscript. No additional external funding received for this study.

## Conflict of Interest

The authors declare that the research was conducted in the absence of any commercial or financial relationships that could be construed as a potential conflict of interest.

## Supplementary Material

Supplementary Figure 1 Calculation of the Naples prognostic score.

Supplementary Figure 2 Study design. AEG, adenocarcinoma of the gastroesophageal junction.

Supplementary Figure 3
**(A)** Relationship between NPS and the OS of patients with stage I AEJ. **(B)** Relationship between NPS and the RFS of patients with stage I AEJ. **(C)** Relationship between NPS and the OS of patients with stage II AEJ. **(D)** Relationship between NPS and the RFS of patients with stage II AEJ. OS, overall survival. RFS, relapse-free survival. AEG, adenocarcinoma of the gastroesophageal junction. NPS, naples prognostic score.

Supplementary Figure 4
**(A)** overall survival curves according to the preoperative ALB. **(B)** overall survival curves according to the preoperative TC. **(C)** overall survival curves according to the preoperative NLR. **(D)** overall survival curves according to the preoperative LMR. ALB, albumin. TC, total cholesterol. NLR, neutrophil-to-lymphocyte ratio. LMR, lymphocyte-to-monocyte ratio.

Supplementary Figure 5
**(A)** Kaplan–Maier curves of overall survival for each SIS group. **(B)** Kaplan–Maier curves of relapse-free survival for each SIS group. SIS, systemic inflammation score.

Supplementary Figure 6
**(A)**, relapse-free survival curves according to the preoperative ALB. **(B)** relapse-free survival curves according to the preoperative TC. **(C)** relapse-free survival curves according to the preoperative NLR. D, relapse-free survival curves according to the preoperative LMR. ALB, albumin. TC, total cholesterol. NLR, neutrophil-to-lymphocyte ratio. LMR, lymphocyte-to-monocyte ratio.
