# Supplementary figures and images for: Prognostic Importance of the Preoperative Naples Prognostic Score for Patients With Adenocarcinoma of the Esophagogastric Junction
Source: Front Oncol. 2020 Dec 16;10:595793. doi: 10.3389/fonc.2020.595793 (PMC7772432; doi:10.3389/fonc.2020.595793)

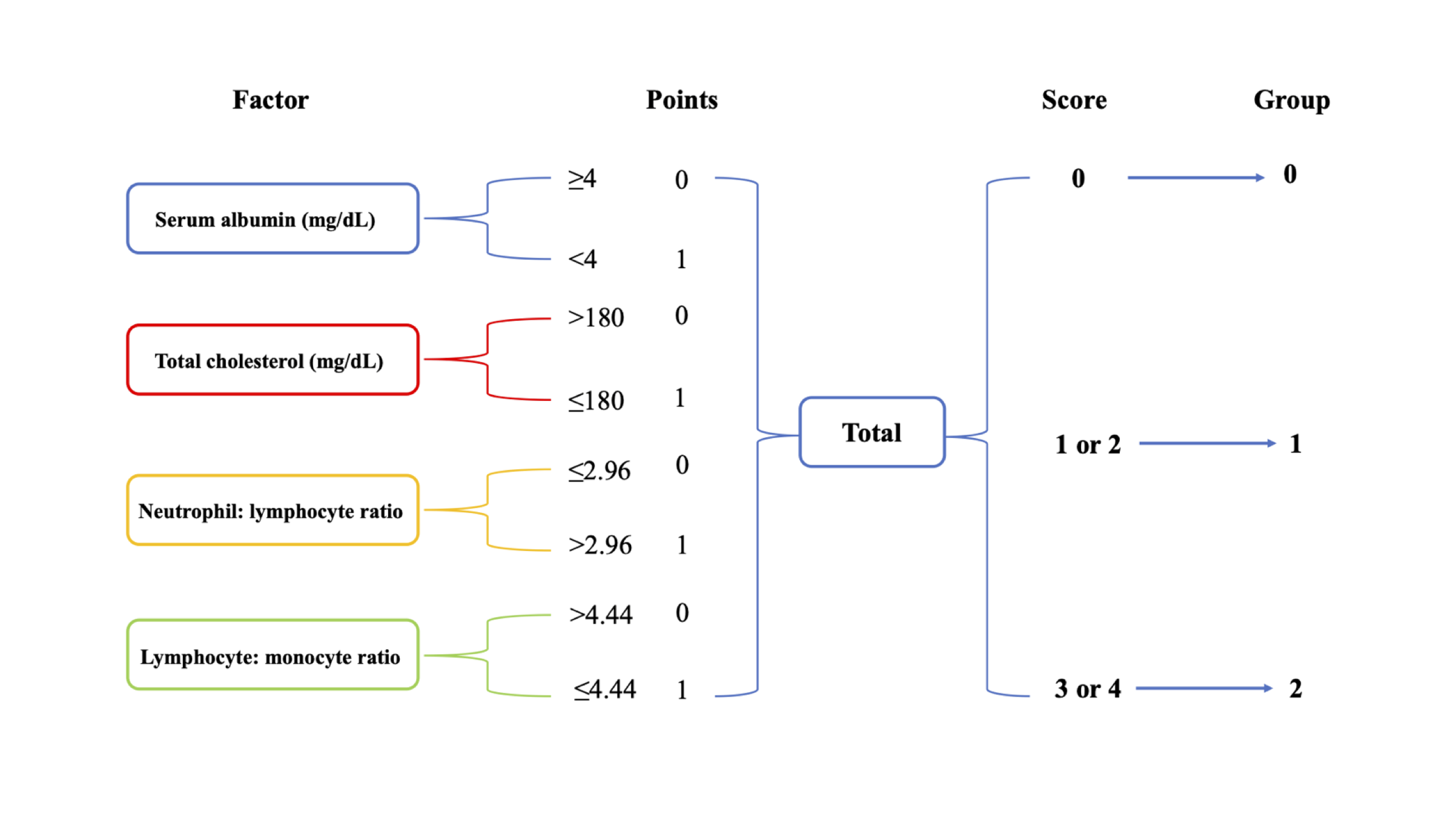

Supplement: Supplementary Figure 1 — Calculation of the Naples prognostic score. [file Image_1.tiff]

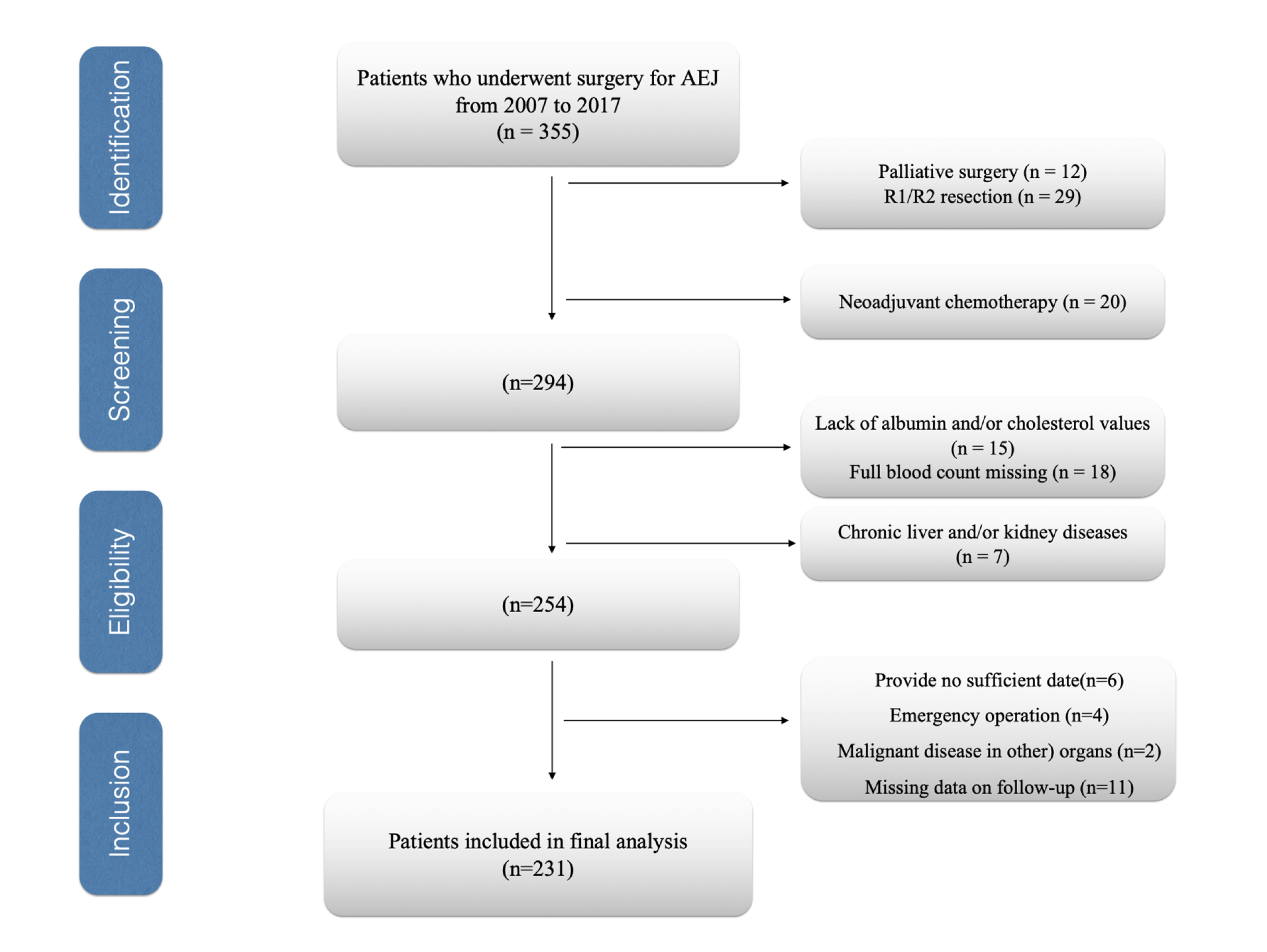

Supplement: Supplementary Figure 2 — Study design. AEG, adenocarcinoma of the gastroesophageal junction. [file Image_2.tiff]

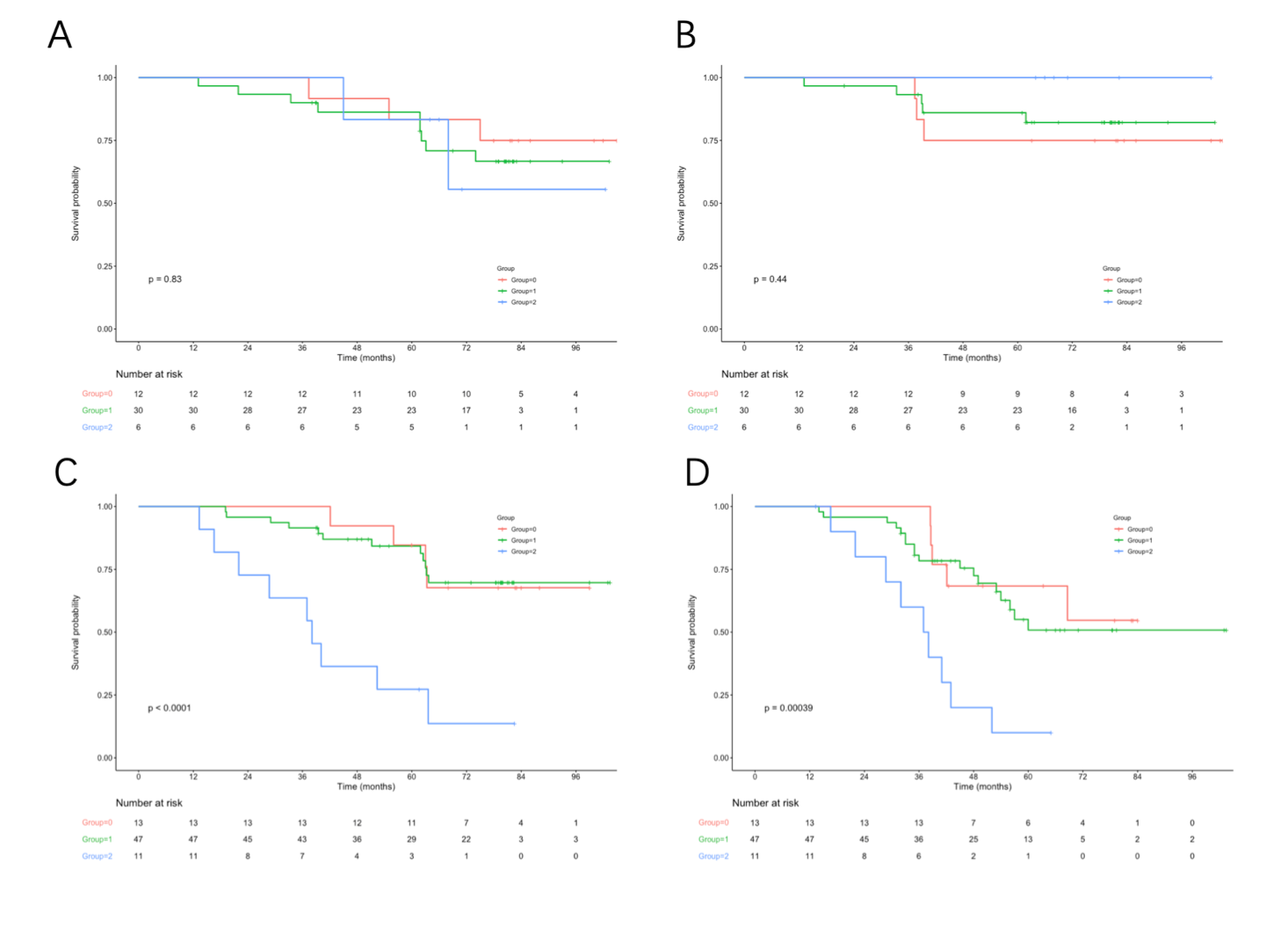

Supplement: Supplementary Figure 3 — (A) Relationship between NPS and the OS of patients with stage I AEJ. (B) Relationship between NPS and the RFS of patients with stage I AEJ. (C) Relationship between NPS and the OS of patients with stage II AEJ. (D) Relationship between NPS and the RFS of patients with stage II AEJ. OS, overall survival. RFS, relapse-free survival. AEG, adenocarcinoma of the gastroesophageal junction. NPS, naples prognostic score. [file Image_3.tiff]

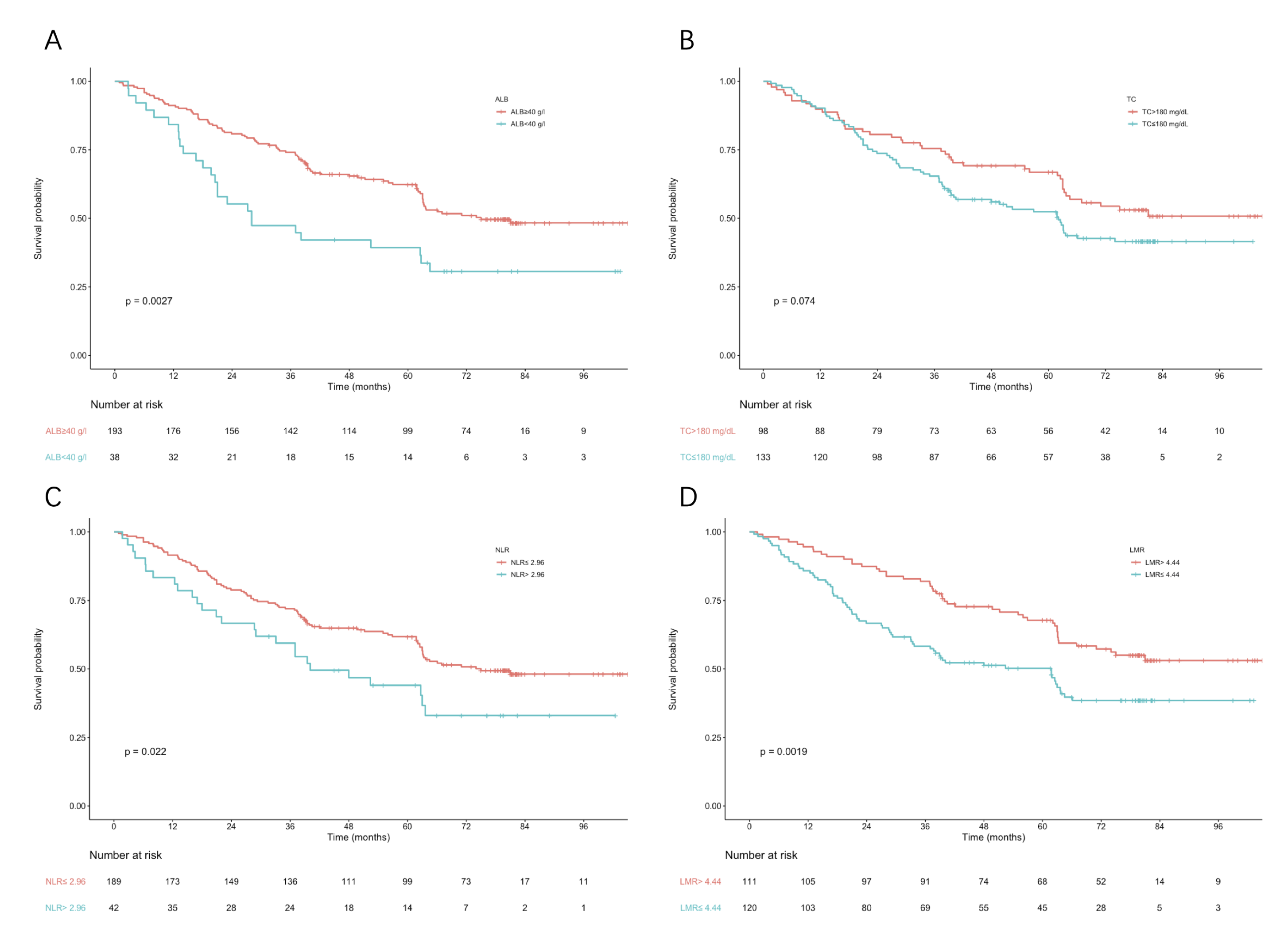

Supplement: Supplementary Figure 4 — (A) overall survival curves according to the preoperative ALB. (B) overall survival curves according to the preoperative TC. (C) overall survival curves according to the preoperative NLR. (D) overall survival curves according to the preoperative LMR. ALB, albumin. TC, total cholesterol. NLR, neutrophil-to-lymphocyte ratio. LMR, lymphocyte-to-monocyte ratio. [file Image_4.tiff]

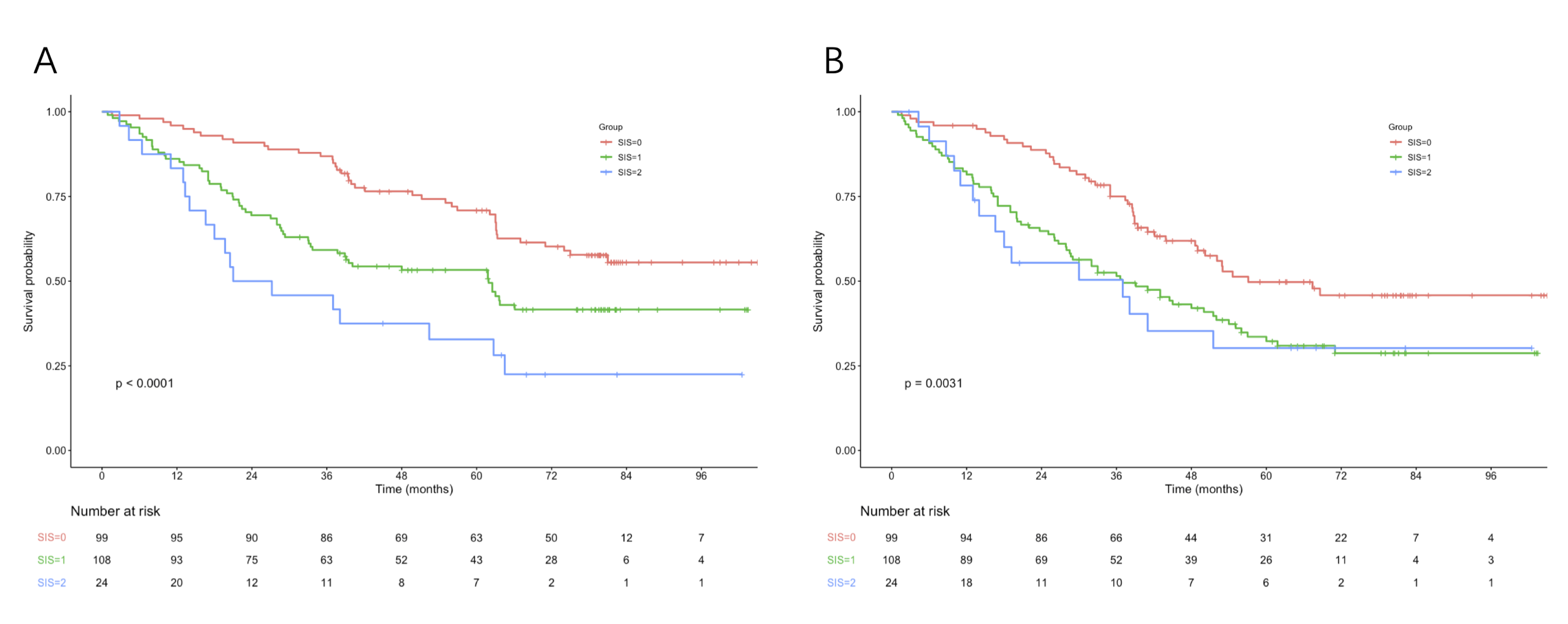

Supplement: Supplementary Figure 5 — (A) Kaplan–Maier curves of overall survival for each SIS group. (B) Kaplan–Maier curves of relapse-free survival for each SIS group. SIS, systemic inflammation score. [file Image_5.tiff]

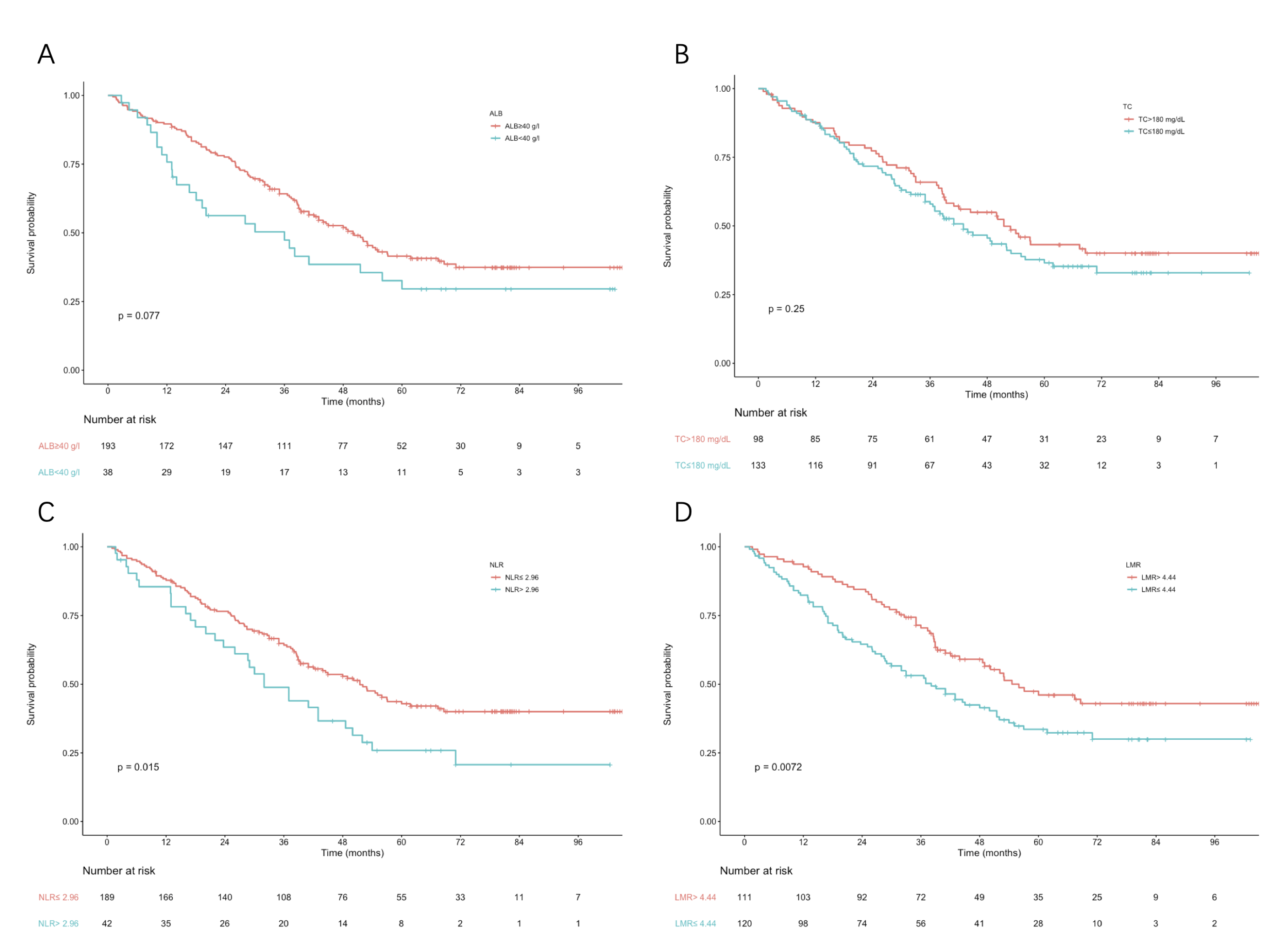

Supplement: Supplementary Figure 6 — (A), relapse-free survival curves according to the preoperative ALB. (B) relapse-free survival curves according to the preoperative TC. (C) relapse-free survival curves according to the preoperative NLR. D, relapse-free survival curves according to the preoperative LMR. ALB, albumin. TC, total cholesterol. NLR, neutrophil-to-lymphocyte ratio. LMR, lymphocyte-to-monocyte ratio. [file Image_6.tiff]
